# Re-Examining High-Fat Diets for Sports Performance: Did We Call the ‘Nail in the Coffin’ Too Soon?

**DOI:** 10.1007/s40279-015-0393-9

**Published:** 2015-11-09

**Authors:** Louise M. Burke

**Affiliations:** Sports Nutrition, Australian Institute of Sport, Canberra, ACT Australia; Mary MacKillop Institute for Health Research, Australian Catholic University, Melbourne, VIC Australia

## Abstract

During the period 1985–2005, studies examined the proposal that adaptation to a low-carbohydrate (<25 % energy), high-fat (>60 % energy) diet (LCHF) to increase muscle fat utilization during exercise could enhance performance in trained individuals by reducing reliance on muscle glycogen. As little as 5 days of training with LCHF retools the muscle to enhance fat-burning capacity with robust changes that persist despite acute strategies to restore carbohydrate availability (e.g., glycogen supercompensation, carbohydrate intake during exercise). Furthermore, a 2- to 3-week exposure to minimal carbohydrate (<20 g/day) intake achieves adaptation to high blood ketone concentrations. However, the failure to detect clear performance benefits during endurance/ultra-endurance protocols, combined with evidence of impaired performance of high-intensity exercise via a down-regulation of carbohydrate metabolism led this author to dismiss the use of such fat-adaptation strategies by competitive athletes in conventional sports. Recent re-emergence of interest in LCHF diets, coupled with anecdotes of improved performance by sportspeople who follow them, has created a need to re-examine the potential benefits of this eating style. Unfortunately, the absence of new data prevents a different conclusion from being made. Notwithstanding the outcomes of future research, there is a need for better recognition of current sports nutrition guidelines that promote an individualized and periodized approach to fuel availability during training, allowing the athlete to prepare for competition performance with metabolic flexibility and optimal utilization of all muscle substrates. Nevertheless, there may be a few scenarios where LCHF diets are of benefit, or at least are not detrimental, for sports performance.

## Key Points

**Table:** 

The current interest in low carbohydrate high fat (LCHF) diets for sports performance is based on enthusiastic claims and testimonials rather than a strong evidence base. Although adaptation to a LCHF (whether ketogenic or not) increases the muscle’s capacity to utilize fat as an exercise substrate, there is no proof that this leads to a clear performance advantage. In fact, there is a risk of impairing the capacity for high intensity exercise.
The current guidelines for carbohydrate intake in the athlete’s training diet appear to be poorly understood. Sports nutrition experts do not promote a “high carbohydrate diet” for all athletes. Rather, the evolving model is that athletes should follow an individualized approach, whereby carbohydrate intake is periodized throughout the training cycle according to the fuel needs of each workout, the importance of performing well in the session and/or the potential to amplify the adaptive response to exercise via exposure to low carbohydrate availability. There is a need for ongoing research and practice to identify a range of approaches to optimal training and competition diets according to the specific requirements of an event and the experience of the individual athlete.

## Introduction

In 2006, after ~15 years of failed attempts to harness adaptations to a high-fat diet as an ergogenic strategy for sports performance in well-trained competitors, this author and a colleague were invited to contribute a commentary on the publication of a new study from the University of Cape Town [1]. After careful inspection of the paper, we speculated on its role as ‘the nail in the coffin’ of fat adaptation for athletic performance [2]. We wrote about what is now known as low-carbohydrate, high-fat (LCHF) diets, “… it seems that we are near to closing the door on one application of this dietary protocol. Scientists may remain interested in the body’s response to different dietary stimuli, and may hunt for the mechanisms that underpin the observed changes in metabolism and function. However, those at the coal face of sports nutrition can delete ‘fat loading’ and high-fat diets from their list of genuine ergogenic aids for endurance and ultra-endurance sports—at least for the conventional events within these categories” [2].

A decade later, theories and claims that fat adaptation can enhance sports performance have strongly re-emerged from several sources via peer-reviewed literature [3–6], lay publications [7], and a highly developed information network that did not exist during the previous incarnation of this dietary theory: social media [8, 9]. Because of the number and fervor of the discussions and the rapidity/reach of the information spread among both scientific and athletic circles, there is a need to re-examine the proposal that an LCHF diet enhances sports performance in competitive athletes. This review summarizes the theory and the evidence to support LCHF diets for athletic performance. It reviews experimental data that informed the conclusions made by this author in 2006 and the context of competitive sport to which they were applied. It then frames the current claims made for the LCHF diet and athletic performance against the current sports nutrition guidelines and any additional evidence against which they should be judged. Finally, it provides a judgement about whether there is justification to recommend the LCHF diet for athletic performance, overall or in specific scenarios, and the research that should be undertaken to continue to evolve the guidelines for the optimal training/competition diet. To provide objectivity in discussing the current promotion of the LCHF diet for enhanced sports performance, quotes from key proponents taken from both peer-reviewed literature and less formal sources are presented. While the inclusion of the latter sources in a scientific review may be considered unconventional, it is now recognized that many scientists actively use social media to promote their views [10] and even conduct research [11], albeit involving non-traditional methodologies. Therefore, it provides an important source of information for constructing the theories that need to be examined. In addition, although the examination of current evidence is primarily based on peer-reviewed literature involving well-controlled scientific trials in trained individuals [12], consideration will be given to anecdotal accounts provided via lay sources to guide future research efforts or identify scenarios in which LCHF diets appear to have utility.

## Sports Performance: A Brief Overview of Fuel Systems

Although it is beyond the scope of this review to adequately summarize the determinants of effective training and optimal competition performances, several general comments related to fueling strategies for training and competition are provided to add context to discussions in this review. Sporting events last from seconds (e.g., jumps, throws) to weeks (e.g., Tour de France cycling stage race), with success being determined by a complex and often changing range of characteristics, including power, strength, endurance, agility, skill, and decision making. The role of training is to accumulate adaptations in the muscle and other body organs/systems to achieve specific characteristics that underpin success in the athlete’s event via a series of systematic and periodized stimuli involving the interaction of nutrition and exercise [13]. Fueling strategies during this period should also be periodized [14] according to the demands of the session and the relative priorities of training with high intensity/quality, practicing competition nutrition and promoting the adaptive response to the training stimulus (see Table 1). In the competition phase, the key role for nutrition is to address the specific limiting factors that would otherwise cause fatigue or a decrement in performance [15]. In many sporting events, the capacity of body fuel stores to support optimal function of the muscle and central nervous system (CNS) is one such factor.

**Table 1:** Summary of current knowledge and guidelines for optimizing fuel needs for training and competition nutrition

Issue	Current knowledge and guidelines
CHO intake in the training diet	Previous focus on ‘high-CHO diets’ should be replaced by consideration of ‘CHO availability’, in which the daily amount and timing of CHO intake is compared with muscle fuel cost of training: ‘high CHO availability’ = intake providing adequate fuel for training needs, while ‘low CHO availability’ = intake is likely to be associated with CHO depletion [53]
	Daily CHO intake should not be static but should be periodized across training microcycles and macrocycles according to fuel cost of training load and the importance of training with high CHO availability [53]
	When workouts involve high-intensity/volume/quality/technique, the day’s eating patterns should provide high CHO availability [53]
	When workouts involve exercise of lower intensity/quality, it is less important to follow patterns that achieve high CHO availability [53]
	Deliberately manipulating diet/training to exercise with low CHO availability can enhance the adaptive response to the training stimulus, and may be periodized into the training program according to individual goals and experience [14, 59]

Issue	Strategy	Targeted event(s)	Current knowledge and guidelines
Optimizing competition performance by increasing fuel availability (especially to addressing the scenario of limited fuel availability)	Increasing muscle phosphocreatine stores to enhance recovery during period between repeated high-intensity intervals: creatine loading	Stop and go sports: e.g., team sports, racket sports	Likely to be effective in sports/positions in which gradual depletion of phosphocreatine stores is limiting to movement patterns [62] Recommended protocol [63]: Rapid loading: 5 days @ 20 g/day creatine in split doses Slow loading: 30 days @ 3 g/day Maintenance: 3 g/day
	Increasing muscle glycogen stores in day(s) prior to event: CHO loading	Prolonged sustained or intermittent sports (usually >90 min) in which muscle glycogen stores become depleted: e.g., marathon, cycling road races, mid-field positions in some team games	Likely to be effective if event would otherwise deplete muscle glycogen stores, leading to reduction in speed and distance covered [64] Recommended protocol [53]: 36–48 h @ 10–12 g/kg/day CHO + taper
	Increase in muscle/liver glycogen in hours prior to event: pre-event meal	Prolonged sustained or intermittent sports (usually >45 min), especially where pre-exercise muscle/liver glycogen are not optimized by other strategies	Likely to be effective if intake increases CHO availability (increase in liver/muscle glycogen > increase in rate of CHO oxidation during exercise) especially in CHO-limited event [53, 65] Recommended protocol [53]: 1–4 g/kg CHO at 1–4 h pre-event
	Increase in exogenous supply of CHO: intake of CHO just prior to and during event Not needed for metabolic effects in events of more than ~75 min, but may be useful for central effects in events greater than ~45 min	Prolonged sustained or intermittent sports (usually >75 min) in which additional fuel source can replace/spare otherwise limited muscle glycogen stores: e.g., marathon, cycling road races, triathlons, team and racket sports	Likely to be effective if intake provides a readily available CHO supply to the muscle, particularly if muscle glycogen becomes depleted. May also address CNS impairment in events or individuals in which reductions in blood glucose concentrations occur [24, 66] Recommended protocol [53]: 1–2.5 h: 30–60 g/h CHO, >2.5–3 h: up to 90 g/h CHO
		Sustained high-intensity sports (45–75 min) not typically considered to be limited by muscle glycogen stores, e.g., cycling time trial, half marathon	Likely to be effective in enhancing pacing strategy via effect on ‘reward centers’ in brain [61, 67] Recommended protocol [53]: frequent exposure of mouth and oral cavity to CHO, including mouth rinse
	Increase in fatty acid availability: fasting or short-term (1–3 days) high-fat diet	Prolonged sustained or intermittent sports (usually >75 min) in which additional fuel source can replace/spare otherwise limited muscle glycogen stores: e.g., marathon, cycling road races, triathlon, team and racket sports	Typically unable to increase (and may even impair) exercise capacity/performance since enhanced fat oxidation is unable to compensate for low muscle glycogen stores Protocol: not recommended [25, 26]
	Increase in fatty acid availability: high-fat pre-event meal (+heparin) or intralipid infusion		No clear performance benefit despite increased fat oxidation. Use of intralipid infusions and heparin to ensure high fatty acid availability is not practical Protocol: not recommended [25, 26]
	Increase in fatty acid availability: feeding of medium chain triglycerides during exercise		Typically unable to increase (and may even impair) exercise capacity/performance since the large amounts needed to impact fuel metabolism cause gut problems [68] Protocol: not recommended [25, 26]

*CHO* carbohydrate, *CNS* central nervous system

In the muscle, exercise is fueled by an intricate system that integrates the production of adenosine triphosphate (ATP) from a combination of intra- and extra-cellular substrates via pathways that are oxygen dependent (oxidation of fat and carbohydrate) and independent (phosphocreatine system and anaerobic glycolysis). The relative contribution of various substrates to the fuel mix depends on various factors, including the mode, intensity, and duration of exercise, the athlete’s training status, and both recent and longer-term dietary intake [16]. For optimal competition performance, the athlete needs a combination of adequate fuel stores in relation to the demands of his or her event as well as ‘metabolic flexibility’, hereby defined in the context of sports performance as the ability to rapidly and efficiently utilize these pathways to maximize ATP regeneration. Although we lack specific data on the metabolic pathways and substrate use in the majority of competitive sports, technological advances such as the development of power meters and global positioning system units have allowed the collection of information such as power output, heart rate, and movement patterns that indirectly capture the metabolic demands of some events. A key understanding from such data is that the fuel demands of many sports are complex and often misunderstood. An example of particular relevance to this review is that sports such as multi-stage road cycling, triathlons, and marathons are classified as endurance and ultra-endurance events conducted at sub-maximal exercise intensities; in fact, for competitive athletes at least, the terrain, pacing strategies, and tactical elements in these events mean that brief but critical parts of the race that often determine the outcomes (e.g., breakaways, hill climbs, surges, sprint finishes) are conducted at higher and often near maximal pace [17–19]. In addition, for such athletes, even the ‘background’ pace from which these brief spurts are performed in endurance sports such as the marathon requires high exercise economy and a sustained use of very high percentage of maximal aerobic intensity [20]. The fueling of the brain and CNS also needs to be considered, since motor recruitment, perception of effort, pacing strategies, and the execution of skills and decision making are also important in determining performance. Here, the main substrates are blood glucose and glycogen stored in the astrocytes [21, 22], although under certain conditions where blood concentrations of ketone bodies are high, they may provide an additional fuel source [23].

Competition nutrition strategies that can enhance fuel availability are summarized in Table 1 and include strategies that attempt to directly increase the size of a limited muscle store (e.g., loading with creatine or carbohydrate) as well as others that attempt to spare the use of the limited store by providing an alternative substrate. For events greater than ~1 h duration, the focus is on tactics that increase carbohydrate availability for the muscle and brain, since low carbohydrate availability is associated with fatigue via a number of peripheral and central mechanisms [24]. Body fat stores—comprising intramuscular triglyceride (IMTG), blood lipids, and adipose tissue IMTG—represent a relatively abundant fuel substrate even in the leanest of athletes. Although endurance training is known to enhance an athlete’s capacity for fat oxidation during exercise [16], a large body of research over the past 3 decades has been dedicated to exploring ways in which this can be further up-regulated to enhance exercise capacity and sports performance by reducing the reliance on the muscle’s limited glycogen stores and/or the need to consume carbohydrate during the event. As summarized in Table 2 and in several reviews [25, 26], acute tactics to increase free fatty availability by increasing fat intake in the hours or days prior to exercise, or consuming fat during exercise have proved unsuccessful or impractical. Therefore, attention has shifted to chronic tactics that could re-tool the muscle to make better use of fat as an exercise fuel.

**Table 2:** Summary of studies of adaptation to ketogenic low-carbohydrate, high-fat diet on performance of trained individuals

Athletes and study design	LCHF adaptation protocol	Performance protocol	Nutritional status/strategies for performance	Performance advantage with LCHF
Pre 2006				
Well-trained cyclists [30] (*n* = 5 M) Crossover design with order effect (control diet first)	7 days HC (57 % CHO) then 28 days LCHF (fat = 85 % E, CHO = <20 g/day)	Cycling; TTE at 60 % *V*O_2_max	Overnight-fasted + no CHO intake during exercise	No NS difference in TTE between trials (151 vs. 147 min for LCHF and HC). Group data skewed by one participant who increased time to fatigue by 156 % on LCHF trial (Fig. 1)
Post 2006				
Moderately trained off-road cyclists [49] (*n* = 8 M) Crossover design	28 days HC (CHO = 50 % E) LCHF (fat = 70 % E, CHO = 15 %)? truly ketogenic	Cycling; *V*O_2_max test	Not stated	No Mixed results, with small increase in *V*O_2_max (56 vs. 59.2 ml/kg/min for HC and LCHF, *p* < 0.01) but reduction in maximum workload (350 vs. 362 W, *p* = 0.037). Small favorable change in body composition with LCHF (loss of ~1.8 kg with body fat loss from 14.9 to 11.0 % BM, *p* < 0.01)
Elite artistic gymnasts [50] (*n* = 8 M) Crossover design with order effect (control diet second)	30 days HC (CHO = 47 % E, 3.9 g/kg) then 30 days LCHF (fat = 55 % E, CHO <25 g/day) (note protein = 40 % E + added supplements)	Strength exercises: squat jump, countermovement jump, push-ups, reverse grip chin test, legs closed barrier maximum test	Not stated	No No change in strength measurements across either dietary phase—therefore, no impairment of performance measures with LCHF diet. Small favorable change in body composition with LCHF (loss of ~1.5 kg with body fat loss from 7.6 to 5.4 % BM)

*BM* body mass, *CHO* carbohydrate, *E* energy, *HC* high-carbohydrate diet, *LCHF* low-carbohydrate high-fat diet, *M* male, *NS* not significant, *TTE* time to exhaustion, *VO*_*2*_ *max* maximal oxygen uptake

## Chronic Adaptation to High-Fat Diets: Research from 1980 to 2006

In contrast to short-term exposure to an LCHF diet, which reduces exercise capacity by depleting liver and muscle stores of glycogen without producing a compensatory increase in fat oxidation [27, 28], longer-term adherence to this dietary regimen causes a range of adaptations to enhance the breakdown, transport, and oxidation of fat in skeletal muscle [29]. Several different approaches have been investigated.

### Ketogenic High-Fat Diets

**Fig. 1:**
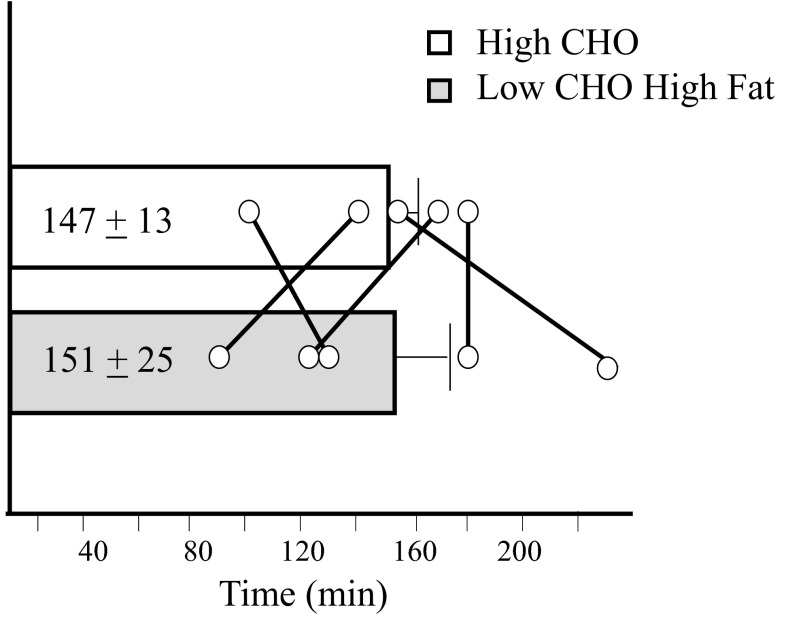
Exercise capacity (time to exhaustion at 62–64 % maximal aerobic capacity, equivalent to ~185 W after 7 days of high-carbohydrate diet followed by 28 days of low-carbohydrate high-fat diet. Data represent mean ± standard error of the mean from five well-trained cyclists (not significantly different), with individual data points represented by *O*. Redrawn from Phinney et al. [30] *CHO* carbohydrate

According to recent reviews [5, 6], historical observations of considerable exercise stamina in explorers who followed traditional Inuit diets almost devoid of carbohydrate (energy contribution: 85 % fat, 15 % protein) led to a laboratory investigation of this phenomenon in the 1980s [30, 31]. In this study by Dr. Stephen Phinney, carefully conducted in a metabolic ward, five well-trained cyclists were tested following 1 week of a carbohydrate-rich diet (~57 % of energy) and again following 28 days of a severely carbohydrate-restricted (<20 g/day) but isoenergetic diet with energy contributions of 85 % fat and 15 % protein (Table 2). This diet was associated with ketosis, as demonstrated by increased blood concentrations of beta-hydroxybutyrate from <0.05 to >1 mmol/L after a week, and this was maintained thereafter. Exercise was monitored by a time to exhaustion cycling test at ~63 % of maximal aerobic capacity (*V*O_2_max) under conditions of low carbohydrate availability (overnight fast and water intake during the ride) [30], with the mean result being a maintenance of exercise capacity (see Fig. 1). Despite the negligible intake of carbohydrate, resting muscle glycogen stores were not depleted but rather reduced to ~45 % of values seen on the high-carbohydrate phase (76 vs. 140 mmol/kg wet weight muscle). Furthermore, in both trials, at the cessation of exercise, muscle glycogen depletion was seen in type 1 fibers with a fourfold reduction in its contribution to fuel use in the LCHF trial. Blood glucose contribution to fuel use was reduced threefold, with gluconeogenic contributions from glycerol released from triglyceride use as well as lactate, pyruvate, and certain amino acids preventing hypoglycemia during exercise as well as allowing glycogen storage between training sessions. Lipid oxidation was increased to make up the fuel substrate for the exercise task.

The researchers’ insights into the results of their study were that “metabolic adaptation to limit CHO [carbohydrate] oxidation can facilitate moderate submaximal exercise during ketosis to the point that it becomes comparable to that observed after a high CHO diet.” Furthermore, they noted that “because muscle glycogen stores require many days for repletion, whereas even very lean individuals maintain appreciable caloric stores as fat, there is potential benefit in this keto-adapted state for athletes participating in prolonged endurance exercise over two or more days”. However, they also commented on the results of *V*O_2_max tests undertaken during each dietary phase with respect to the ketogenic diet: “… the price paid for the conservation of CHO during exercise appears to be a limitation of the intensity of exercise that can be performed … there was a marked attenuation of respiratory quotient [RQ] value at *V*O_2_max suggesting a severe restriction on the ability of subjects to do anaerobic work”. Their explanation for this observation was that “the controlling factor does not appear to be the presence or absence of substrate in the fiber. Rather it is more likely a restriction on substrate mobilization or fiber recruitment. The result, in any case, is a throttling of function near *V*O_2_max”.

The researchers were clear that their ketogenic diet did not, as is popularly believed, enhance exercise capacity/performance, noting that, at best, endurance at sub-maximal intensities was preserved at the expense of ability to undertake high-intensity exercise. However, examination of the design and outcomes call for further caution. Although excellent dietary control was achieved in this study, few details were provided of the training protocols followed by the cyclists. It is curious in light of the order effect in the study design (all subjects undertook the ketogenic exercise trial 4 weeks after their carbohydrate trial), that no benefit to exercise capacity was derived from an additional training period. Furthermore, it should be recognized that the exercise task was undertaken under conditions that should have favored any advantage to being adapted to low carbohydrate availability (moderate-intensity exercise, overnight fast, no intake of carbohydrate during exercise). However, and most importantly, the focus on the mean outcomes of the trial in a small sample size hides the experiences of the individual cyclists. As shown in Fig. 1, the published interpretations of the results of this study are largely skewed by the experience of a single subject who showed a large enhancement of exercise capacity after the ketogenic diet (and additional training period). Indeed, statistical analysis of the same data using a magnitude-based inferences approach [32] reveals an unclear outcome, with the chances of a substantially positive, trivial, and substantially negative outcome being 32, 32, and 36 %, respectively (Stellingwerff, personal communication).

### Non-Ketogenic High-Fat Diets

A number of studies have been undertaken in trained individuals involving exposure for ≥7 days to a diet high in fat and restricted in carbohydrate content without achieving ketosis [33–37]; much of this work was driven by Dr. Vicki Lambert and Professor Tim Noakes from the University of Cape Town. Two studies in which carbohydrate and fat intake was manipulated in trained populations have not been included in this summary since the dietary changes were not sufficient to meet the criteria of >60 % fat intake or <25 % carbohydrate intake [38, 39]. The summarized literature (Table 3) includes one study that focused on titrating the carbohydrate content of the diet in modestly trained female cyclists [33] and four studies that specifically set out to adapt their subjects to a high-fat diet [34–37], although in one case, the smaller degree of carbohydrate restriction resulted in a failure to create clear differences in muscle glycogen content between treatments [37]. Again, the diets provided within studies were isoenergetic and aimed at maintaining energy balance.

**Table 3:** Effect of up to 28 days of adaptation to high-fat low carbohydrate diet on performance of trained individuals

Athletes	LCHF adaptation protocol	Performance protocol	Nutritional status/strategies for performance	Performance advantage with LCHF
Moderately trained cyclists [33] (*n* = 7 F) Crossover design	7 days LCHF (fat = 59 % E, CHO = 1.2 g/kg BM) HC (CHO = 6.4 g/kg BM)	Cycling TTE at 80 %* VO*_2_max	3–4 h after meal, no CHO intake during exercise	No In fact, performance deteriorated with LCHF. Time to exhaustion reduced by 47 % on LCHF trial
Well-trained cyclists [34] (*n* = 5 M) Crossover design	14 days LCHF (fat = 67 % E, CHO = 17 % E^a^) HC (CHO = 74 % E^a^)	Cycling 30 s Wingate test + TTE at 90 % *V*O_2_max + TTE at 60 %* VO*_2_max	Overnight-fasted + no CHO intake during exercise	No: two higher intensity tests Yes: Submaximal cycling Time to exhaustion increased by 87 % on LCHF trial commenced with lower glycogen stores due to preceding exercise
Well-trained cyclists [35] (*n* = 16 M) Parallel-group design	15 days LCHF (fat = 69 %E, CHO = 2.2 g/kg BM) HC (CHO = 5.5 g/kg BM)	Cycling 150 min at 70 %* VO*_2_max + 40 km TT Performance measured at *t* = 0, 5, 10, and 15 days	MCT intake 1.5 h before event (~14 g) MCT (0.3 g/kg/h) and CHO (0.8 g/kg/h) during exercise	No TT performance increased over time in both groups as a result of training protocol. Significant improvements seen in both groups by day 10, but no difference in mean improvement between groups. Important finding of study: adaptations achieved after only 5 days of high-fat diet
Well-trained cyclists [36] (*n* = 7 M) Crossover design	14 days LCHF (fat = 66 % E, CHO = ~2.4 g/kg) HC (CHO = ~8.6 g/kg, 70 % CHO)	Cycling 5 h including 15 min TT + 100 km TT	LCHF = high-fat pre-event meal HC = high CHO pre-event meal Both: 0.8 g/kg/h CHO during ride	Yes: submaximal intensity exercise No: higher-intensity exercise Relative to baseline: HC showed small NS decreases in performance of both 15 min TT and 100 km TT LCHF showed larger but NS decrease in performance of 15 min TT but small NS improvement in 100 km TT
Well-trained duathletes [37] (*n* = 11 M) Crossover design	5 weeks LCHF (fat = 53 % E, CHO = ~3.6 g/kg) HC (CHO = ~6.9 g/kg, 68 % CHO)	Cycling 40 min incremental protocol + 20 min TT @ ~89 %* VO*_2_max Running (separate day) Outdoor 21 km TT	LCHF = high-fat pre-event meal HC = high CHO pre-event meal Intake pre and during half marathon not stated	No Self-selected work output similar for cycling TT in both dietary treatments (298 ± 6 vs. 297 ± 7 W, NS) for LCHF and HC, respectively. Half marathon time not different between trials (80 min 12 s ± 86 s vs. 80 min 24 s ± 82 s, NS)

*BM* body mass, *CHO* carbohydrate, *E* energy *F* female, *HC* high-carbohydrate diet, *LCHF* low-carbohydrate high-fat diet, *M* male, *MCT* medium chain triglyceride, *NS* not significant, *TT* time trial, *TTE* time to exhaustion, *VO*_*2*_ *max* maximal oxygen uptake

^a^g/kg intakes unavailable

In the case of studies specifically focused on adapting athletes to a high fat intake, the rationale of increasing dietary fat involved increasing IMTG stores [37], restricting carbohydrate to reduce muscle glycogen content [34–36] and allowing sufficient exposure for adaptations to occur to retool the muscle to alter fuel utilization patterns during exercise to compensate for altered fuel availability [34–37]. The avoidance of ketosis was chosen to remove its confounding effect on the relationship between respiratory exchange ratio and substrate utilization during exercise, thereby preventing a true measurement of changes in carbohydrate and fat oxidation during exercise [34]. A range of adaptive responses to the LCHF diet was observed or confirmed in the trained individuals.

As summarized in Table 3, the effect of exposure to the LCHF diets on exercise capacity/performance was tested under a range of different exercise scenarios and feeding strategies. This includes a series of exercise protocols undertaken sequentially [34] or within a single exercise task [36], as well as dietary strategies that would either further increase fat availability [33, 36, 37], increase carbohydrate availability [35–37], or deliberately decrease carbohydrate availability against current guidelines or common practices [34]. In some cases, different dietary strategies were implemented before and during the exercise protocols for the high carbohydrate and LCHF trials, making it difficult to isolate the effects of the fat adaptation per se [36, 37]. This variability in study design makes it difficult to make a single and all-encompassing assessment of the effect of LCHF on exercise, as is popularly desired. Theoretically, however, it offers the opportunity to identify conditions under which adaptation to a high-fat diet may be of benefit or harm to sports performance. Unfortunately, the small number of studies and the small sample sizes in the available literature do not allow this opportunity to be fully exploited. The learnings from these studies have been incorporated into the summary at the end of this section. In the meantime, attention is drawn to two important observations from this body of literature:

1. Evidence of reduced utilization of muscle glycogen as an exercise fuel following adaptation to LCHF cannot be considered true glycogen ‘sparing’ since the observations are confounded by lower resting glycogen concentrations, which are known to reduce glycogen use per se [40]. Only scenarios in which muscle glycogen concentrations are matched prior to exercise can allow the specific effect of fat adaptation on muscle glycogen utilization as an exercise fuel to be measured.
2. The period required for adaptation to the non-ketogenic LCHF is shorter than previously considered. According to the time course study of Goedecke et al. [35], whereby muscle fuel utilization was tracked after 5, 10, and 15 days of exposure to the LCHF diet, a substantial shift to increase fat oxidation and reduce carbohydrate utilization was achieved by 5 days without further enhancement thereafter. Of course, it should be noted that a shift in respiratory exchange ratio during exercise, marking shifts in substrate utilization can reflect the prevailing availability of substrate rather than a true adaptation in the muscle.

However, other studies have confirmed the presence of a robust change in the muscle’s substrate use via observations of alterations in the concentrations or activity of proteins or metabolites that regulate fatty acid availability, as well as the persistence of increased fat oxidation in the face of abundant carbohydrate supplies. Such evidence is discussed later.

Importantly, the observation from this series of studies—that retooling of already trained muscle to optimize muscle utilization of fat as an exercise fuel can be achieved in a conveniently short period—led in part to the next phase of investigation, in which attempts were made to enhance sports performance by separately optimizing the muscle’s capacity for lipid and carbohydrate utilization.

### Fat Adaptation and Carbohydrate Restoration

**Fig. 2:**
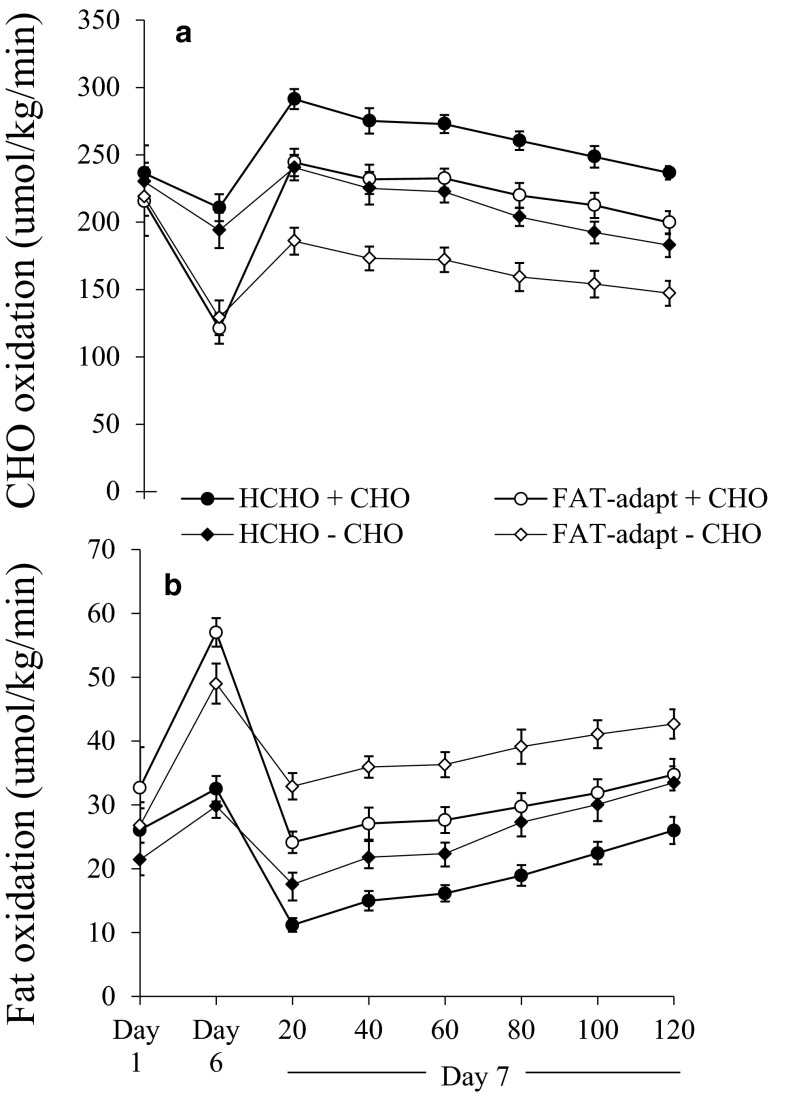
Effect of 5 days of adaptation to a low-carbohydrate high-fat diet and 1 day of a high-carbohydrate diet to restore muscle glycogen (FAT-adapt) on rate of carbohydrate oxidation (**a**) and rate of fat oxidation (**b**) during cycling at 70 % maximal aerobic capacity compared with control trial (6 days of a high-carbohydrate diet). Data are taken from two studies in which no additional carbohydrate was consumed on the day of a 120-min cycling bout at this same workload (−carbohydrate) [45] or where carbohydrate was consumed before and throughout the 120-min cycling task (+carbohydrate) [41]. Values are mean ± SEM for eight well-trained cyclists at day 1 (baseline), day 6 (after 5 days of low-carbohydrate high-fat diet or 5 days of high-carbohydrate diet) and during 120 min of steady-state cycling on day 7 (following 1 day of high-carbohydrate diet). The adaptation to 5 days of high-fat diet increased fat utilization and reduced carbohydrate utilization during submaximal exercise, persisting despite the restoration of muscle glycogen on day 6 or the intake of additional carbohydrate before/during exercise on day 7. Reproduced from Burke et al. [41] with permission. *CHO* carbohydrate, *HCHO* high carbohydrate

In the absence of finding clear benefits from adapting to a high-fat diet on exercise performance, attention was drawn to a tactic of dietary periodization in which a short-term adaptation to an LCHF diet might be followed by glycogen restoration (‘carbohydrate loading’) with 1–3 days of a carbohydrate-rich diet with [1, 36, 41–44] or without [45] additional carbohydrate intake pre- and during subsequent exercise. Such strategies were aimed at promoting simultaneous increases in fat and carbohydrate availability and utilization during exercise. Indeed, studies that directly compared fuel utilization during submaximal exercise under controlled conditions after the fat adaptation protocol and then again after carbohydrate restoration practices [41, 42, 45] showed that the muscle re-tooling was robust enough to maintain an increase in fat utilization during exercise in the face of the practices that supported plentiful carbohydrate availability (Fig. 2).

As discussed in the previous section, a range of permutation and combinations of dietary strategies and exercise protocols can be investigated in combination with the fat adaptation and carbohydrate restoration strategies to test the effect of such dietary periodization on exercise capacity/performance. The available literature is summarized in Table 4 and includes multiple studies from the author’s own laboratory as well as from the University of Cape Town. However, within this group of investigations, only one fully published study [1] attempted to investigate an exercise test that bears any real resemblance to a sporting competition; its characteristics include a sole focus on performance rather than a hybrid of metabolism and performance, self-pacing, and a protocol interspersing passages of high-intensity exercise against a background of moderate-intensity work to reflect the stochastic profile of many real-life events. This study [1], which prompted the 2006 editorial about which this review revolves, merits special reflection before a general summary of the literature is provided.

**Table 4:** Effect of adaptation (5–10 days) to high-fat low-carbohydrate diet followed by carbohydrate restoration in trained individuals

Participant characteristics	LCHF adaptation protocol	CHO restoration	Performance protocol	Nutritional status/strategies for performance	Performance advantage with LCHF adaptation + CHO restoration
Well-trained cyclists/triathletes [45] (*n* = 8 M) Crossover design	5 days LCHF-adapt (fat = 68 % E; CHO = 18 % E, 2.5 g/kg BM) or HC (CHO = 74 % E, 9.6 g/kg BM CHO)	1 day rest + high CHO (CHO = 75 % E, 10 g/kg BM)	Cycling 120 min at 70 % *V*O_2_max + ~30 min TT (time to complete 7 J/kg BM)	Fasted + no CHO intake during exercise	Perhaps for individuals Two participants performed badly on HC trial, probably because of hypoglycemia. Plasma glucose better maintained on LCHF-adapt trial. TT not significantly different between trials: 30.73 ± 1.12 vs. 34.17 ± 2.62 min for LCHF and HC trial. However, mean difference in TT = 8 % enhancement with LCHF trial (*p* = 0.21, NS; 95 % CI –6 to 21).
Well-trained cyclists and triathletes [41] (*n* = 8 M) Crossover design	5 days LCHF-adapt (fat = 68 % E; CHO = 18 % E, 2.5 g/kg BM) or HC (CHO = 70 % E, 9.3 g/kg BM CHO)	1 day rest + high CHO (CHO = 75 % E, 10 g/kg BM)	Cycling 120 min at 70 % *V*O_2_max + ~30 min TT (time to complete 7 J/kg BM)	CHO intake 2 h before exercise (2 g/kg BM) and during exercise (0.8 g/kg/h)	No Plasma glucose maintained in both trials due to CHO intake during exercise. Difference in TT between trials was trivial: LCHF-adapt = 25.53 ± 0.67 min; HC = 25.45 ± 0.96 min (*p* = 0.86, NS). Mean difference in TT = 0.7 % impairment with LCHF-adapt trial (95 % CI –1.7 to 0.4)
Highly-trained cyclists and triathletes [42] (*n* = 7 M) Crossover design	6 days LCHF-adapt (fat = 69 % E CHO = 16 % E, 2.5 g/kg BM) or HC (CHO = 75 % E, 11 g/kg BM)	1 day rest + high CHO (CHO = 75 % E, 11 g/kg BM)	Cycling 240 min at 65 % *V*O_2_max + 60 min TT (distance in 1 h)	CHO intake before exercise (3 g/kg BM) and during exercise (1.3 g/kg/h)	No or perhaps for individuals TT performance NS between trials: 44.25 ± 0.9 vs. 42.1 ± 1.2 km for LCHF-adapt and HC trial. However, mean difference in TT performance = 4 % enhancement with LCHF-adapt (*p* = 0.11, NS) (95 % CI –3 to 11)
Highly-trained cyclists and triathletes [43] (*n* = 7 M) Crossover design	5 days LCHF-adapt (fat = 69 % E CHO = 16 % E, 2.5 g/kg BM) or HC (CHO = 75 % E, 11 g/kg BM)	1 day rest + high CHO (CHO = 75 % E, 11 g/kg BM)	Cycling 240 min at 65 % *V*O_2_max + 60 min TT (distance in 1 h)	CHO intake before exercise (3 g/kg BM) and during exercise (1.3 g/kg/h)	No Additional six subjects undertaken to test for Type 1 error in previous study [42]. TT performance NS between trials: 42.92 ± 1.46 vs. 42.94 ± 1.41 km for LCHF-adapt and HC trial (*p* = 0.98). Performance difference = 0.02 km or 0.1 %
Trained cyclists and triathletes [44] (*n* = 5 M) Crossover design	10 days LCHF-adapt (fat = 65 % E, CHO = 15 % E, 1.6 g/kg BM) or HC (CHO = 53 % E, 5.8 g/kg BM)	3 days high CHO (CHO = 65 % E, 7 g/kg BM) + 1 day rest	Cycling 150-min cycling at 70 % *V*O_2_max + 20-km (~30 min) TT	MCT intake 1 h before event (~14 g); MCT (0.3 g/kg/h) and CHO (0.8 g/kg/h) during exercise	Yes Difference in TT performance = 4 % enhancement with LCHF-adapt: 29.35 ± 1.25 vs. 30.68 ± 1.55 min for LCHF-adapt and HC (*p* < 0.05)
Well-trained cyclists [36] (*n* = 7 M) Crossover design	11.5 days LCHF-adapt (~2.4 g/kg, 15 % CHO; 66 % fat) or HC (CHO = ~8.6 g/kg, 70 % E)	2.5 days high CHO (6.8 g/kg BM)	Cycling 5-h protocol including 15-min TT + 100-km TT	HC: High-CHO pre-event meal Both: 0.8 g/kg/h CHO during exercise	Perhaps—submaximal intensity exercise No—higher-intensity exercise Relative to baseline testing: HC trial showed small NS decrease in performance of both 15-min TT and 100-km TT. LCHF-adapt showed no change in 15-min TT but small NS enhancement of 100-km TT
Well-trained cyclists [1] (*n* = 8 M) Crossover design	6 days LCHF-adapt (fat = 68 % E CHO = 17 % E, 1.8 g/kg BM) or HC (CHO = 68 % E, 7.5 g/kg BM)	1 day rest + high CHO (8–10 g/kg)	Cycling 100 km TT, including 4 × 4-km sprints + 5 × 1-km sprints	CHO consumed during ride	No—in fact, performance enhancement of 1-km sprints Differences between 100-km TT performances: NS (156 min 54 s vs. 153 min 10 s for LCHF-adapt vs. HC). Difference between power output during 4-km sprints: NS. However, power during 1-km sprints (undertaken at >90 % PPO) was significantly reduced in LCHF-adapt trial

All values are mean ± standard error of the mean

*BM* body mass, *CHO* carbohydrate, *CI* confidence interval, *E* energy, *HC* high carbohydrate, *LCHF* low-carbohydrate high-fat diet, *M* male, *MCT* medium-chain triglyceride, *NS* not significantly different, *PPO* peak power output, *TT* time trial, *VO2max* maximal oxygen uptake

**Fig. 3:**
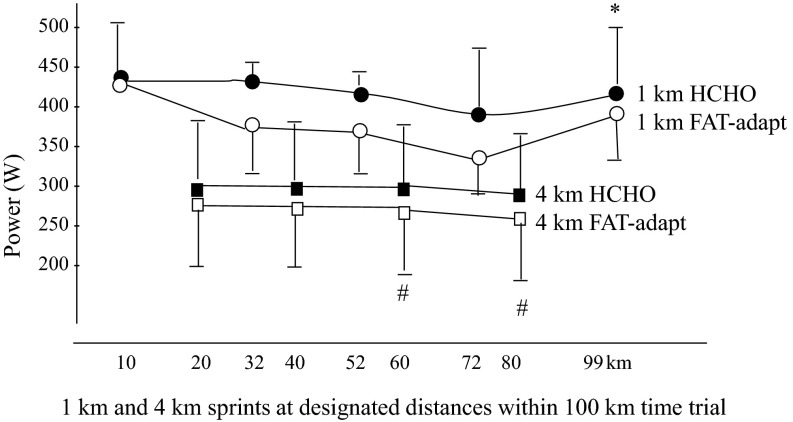
Power outputs during 1- and 4-km sprints undertaken within a 100-km self-paced cycling time trial after a 6-day high-carbohydrate diet and 5 days of a low-carbohydrate high-fat diet followed by 1 day of a high-carbohydrate diet (fat-adapt) [1]. 100-km total time: 153:10 vs. 156:54 min for carbohydrate vs. FAT-adapt, not significant. Values are means ± standard deviation for eight well-trained cyclists. Power outputs decreased over time in both trials with 4-km sprints (^#^ *p* < 0.05), but did not differ between trials. However, with the 1-km sprints, mean power was significantly lower after the fat-adaptation treatment (Fat-adapt) compared with the high-carbohydrate diet (*p < 0.05). Reproduced from Havemann et al. [1] with permission. *HCHO* high carbohydrate

Havemann et al. [1] had well-trained cyclists undertake either a 6-day LCHF diet followed by a 1-day high-carbohydrate diet or 7 days of high-carbohydrate diet before undertaking a laboratory-based cycling protocol designed to test some of the features of endurance sporting events. Specifically, cyclists were required to undertake a series of sprints throughout the self-paced 100-km trial: 4-km sprints undertaken at ~78–84 % peak power output and 1-km sprints undertaken at >90 % peak power output (see Fig. 3). Overall, differences in the performance times for the 100-km time trial (TT) were not statistically significant, although the mean performance on the high-carbohydrate trial was 3 min 44 s or ~2.5 % faster (153 min, 10 s for high-carbohydrate trial and 156 min, 53 s for LCHF adapted, *p* = 0.23). While there was no difference between trials with regard to the 4-km sprint times, performance of the 1-km sprints was significantly impaired in the LCHF-adapted trial in all subjects, including the three subjects whose overall 100-km TT performance was faster than in their high-carbohydrate trial. The authors stated that although adaptation to the LCHF diet followed by carbohydrate restoration increased fat oxidation during exercise, “it reduced high-intensity sprint power performance, which was associated with increased muscle recruitment, effort perception and heart rate”.

**Fig. 4:**
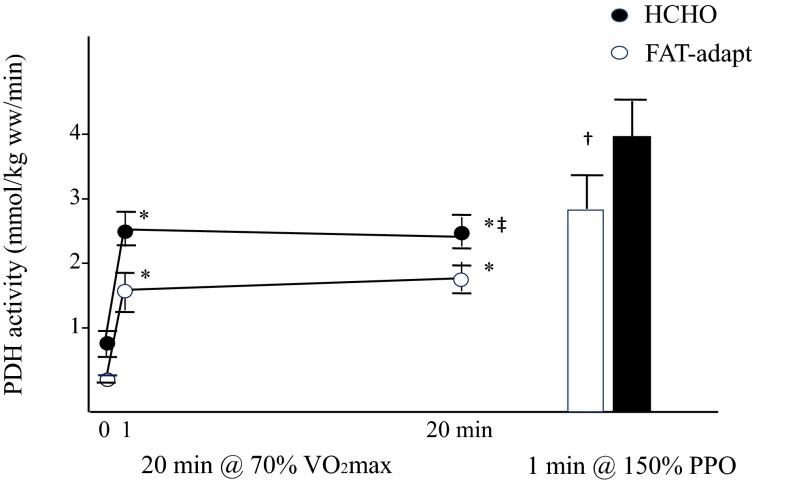
Pyruvate dehydrogenase activity in the active form at rest, during 20 min of cycling at ~70 % maximal aerobic capacity followed by a 1-min sprint at 150 % of peak power output after either a 5-day adaptation to a low-carbohydrate high-fat diet followed by a 1-day high-carbohydrate diet (FAT-adapt) or 6 days of a high-carbohydrate diet. Values are means ± standard error of the mean for seven well-trained cyclists. *Different from 0 min, ^‡^trial effect: HCHO trial > FAT-adapt trial; ^†^time point: HCHO trial > FAT-adapt where significance is set at *p* < 0.05. Reproduced from Stellingwerff et al. [46] with permission. *HCHO* high carbohydrate, *PDH* pyruvate dehydrogenase, *PPO* peak power output, *VO*_*2*_ *max* maximal aerobic capacity

Although the mechanisms associated with the compromised performance in this study were unclear, speculations by the authors included “increased sympathetic activation, or altered contractile function and/or the inability to oxidize the available carbohydrate during the high intensity sprints”. Indeed, evidence for this latter suggestion was provided by data from this author’s own laboratory collected contemporaneously. In an investigation of possible mechanisms to explain the performance outcomes associated with the LCHF-adaptation and carbohydrate-restoration model, we examined muscle metabolism at rest, during sub-maximal exercise, and after an all-out 1-min sprint following the usual dietary treatment (Fig. 4) [46]. In comparison with the control trial (high-carbohydrate diet), we found that adaptation to the LCHF diet and subsequent restoration of muscle glycogen was associated with a reduction in glycogenolysis during exercise, and a reduction in the active form of pyruvate dehydrogenase (PDHa) at rest, during submaximal cycling, and during sprint cycling. Explanations for the down-regulated activity of this enzyme complex responsible for linking the glycolytic pathway with the citric acid cycle included the observed post-sprint decrease in concentrations of free adenosine monophosphate (AMP) and adenosine diphosphate (ADP) and potentially an up-regulation of PDH kinase (PDK) activity, which has previously been observed in association with a high-fat diet [47]. This study provided evidence of glycogen ‘impairing’ rather than ‘sparing’ in response to adaptation to an LCHF diet and a robust explanation for the impairment of key aspects of exercise performance as a result of this dietary treatment.

### Summary of Learnings from the Literature: 1999–2006

Key interpretations by this author from the literature on adaptation to an LCHF conducted up until 2006 are summarized below:

1. Exposure to an LCHF diet in the absence of ketosis causes key adaptations in the muscle in as little as 5 days to retool its ability to oxidize fat as an exercise substrate. Adaptations include, but are not limited to, an increase in IMTG stores, increased activity of the hormone-sensitive lipase (HSL) enzyme, which mobilizes triglycerides in muscle and adipose tissue, increases in key fat-transport proteins such as fatty acid translocase [FAT-CD36] and carnitine-palmitoyl transferase (CPT) (for extended review, see Yeo et al. [29]). Together, these adaptations further increase the already enhanced capacity of the aerobically trained muscle to utilize endogenous and exogenous fat stores to support the fuel cost of exercise of moderate intensity. Rates of fat oxidation during exercise may be doubled by fat-adaptation strategies.
2. These muscle-retooling activities stimulated by fat adaptation are sufficiently robust that they persist in the face of at least 36 h of aggressive dietary strategies to increase carbohydrate availability during exercise (e.g., glycogen supercompensation, pre-exercise carbohydrate intake, high rates of carbohydrate intake during exercise). Although the increased carbohydrate availability reduces rates of fat oxidation compared with fat adaptation alone, fat utilization remains similarly elevated above comparative rates in the absence of fat adaptation. The time course of the ‘washout’ of retooling is unknown.
3. In addition to up-regulating fat oxidation at rest and during exercise, exposure to an LCHF diet down-regulates carbohydrate oxidation during exercise. Direct [34, 42, 45] and indirect [45] techniques of measuring the source of changes in substrate utilization show that changes in utilization of muscle glycogen, rather than blood glucose or exogenous glucose, account for the change in carbohydrate use. The reduction in glycogen use persists in the face of glycogen supercompensation [45] and high-intensity exercise [46], noting that it is robust and independent of substrate availability. A down-regulation of PDH activity explains at least part of the impairment of glycogen utilization as an exercise fuel [46], representing a decrease in metabolic flexibility.
4. Despite the enhanced capacity for utilization of a relatively limitless fuel source as an exercise substrate, fat-adaptation strategies with or without restoration of carbohydrate availability do not appear to enhance exercise capacity or performance per se. Several inter-related explanations are possible for the failure to observe benefits:
  Type II statistical error: failure to detect small but important changes in performance due to small sample sizes [34], individual responses [42, 45], and poor reliability of the performance protocol. While this explanation often looks attractive [43], in some cases, further exploration and enhanced sample size increases confidence in the true absence of a performance enhancement [43].
  Benefits are limited to specific scenarios: characteristics of conditions under which fat-adaptation strategies appear to be more likely to be beneficial include protocols of prolonged sub-maximal exercise in which pre-exercise glycogen is depleted and/or no carbohydrate is consumed during exercise (e.g., low-carbohydrate availability).
  Benefits are limited to specific individuals: characteristics of individuals who may respond to fat-adaptation strategies include carbohydrate-sensitive individuals who are subjected to scenarios in which carbohydrate cannot be consumed during exercise.
5. The experience of athletes, at least in the short-term exposure to LCHF diets, is of a reduction in training capacity and increase in perceived effort, heart rate, and other monitoring characteristics, particularly in relation to high-intensity/quality training, which plays a core role in a periodized training program [40].
6. Fat-adaptation strategies may actually impair exercise performance, particularly involving shorter high-intensity events or high-intensity phases during a longer event, which require power outputs or intensities of 85–90 % maximum level or above. This is likely to be due to the impairment of the muscle glycogen utilization needed to support high work rates, even in scenarios where strategies to achieve high carbohydrate availability are employed.

On the basis that conventional competitive sports generally provide opportunities to achieve adequate carbohydrate availability, that fat-adaptation strategies reduce rather than enhance metabolic flexibility by reducing carbohydrate availability and the capacity to use it effectively as an exercise substrate, and that athletes would be unwise to sacrifice their ability to undertake high-quality training or high-intensity efforts during competition that could determine the outcome of even an ultra-endurance sport, this author decided to abandon a research and practical interest in fat-adaptation strategies. A meta-analysis published about the same time on the effect of the carbohydrate and fat content of athletic diets on endurance performance [48] summarized that the heterogeneity around their findings that high-carbohydrate diets (defined as >50 % of energy from carbohydrate) have a moderate (effect size 0.6) benefit on exercise capacity compared with high-fat diets (defined as >30 % of energy from fat) showed that “a conclusive endorsement of a high-carbohydrate diet is hard to make”. However, this heterogeneity speaks to the limitations of undertaking a meta-analysis with such a broad and undefined theme as well as the problem of the ‘black and white’ thinking that is discussed in the conclusion to this review.

## Update on Fat Adaptation Literature Since 2006

Given the recent escalation in the promotion of LCHF diets for sports performance, it could be assumed that the last decade has seen the publication of a considerable number of studies with clear evidence of benefits to sports performance following the implementation of fat-adaptation strategies. Yet, to the knowledge of this author, only two new investigations of LCHF diets in athletes have appeared in the peer-reviewed literature since 2006 [49, 50]. These studies, summarized in Table 2, fail to show performance benefits associated with a ketogenic LCHF diet, although there is evidence of a small but favorable reduction in body fat levels. Nevertheless, there are some peculiarities with the design or methodologies of these studies, including the failure of one study to achieve the carbohydrate restriction typically associated with the ketogenic LCHF diet, and they have failed to become widely cited, even by supporters of the LCHF movement. Rather, the current interest in chronic application of LCHF eating by athletes appears to be driven by enthusiastic discussion in lay and social media by (mostly) non-elite athletes of sporting success following experimentation with such diets as well as a range of outputs from several sports scientists who are researchers and advocates of this eating style [3–8]. It is uncertain whether there is a cause–effect relationship between these sources (or the direction of any relationship), but the fervor merits attention. In the absence of compelling new data, the reader is alerted to several elements in the discussions that are positive and some that are concerning:

1. Peer-reviewed publications from the key scientific protagonists of the LCHF movement [3, 5, 6] generally show measured and thoughtful insights, based on a re-examination of previously conducted studies, personal experiences, anecdotal observations from the sports world, and the general interest in tackling modern health problems with the LCHF approach [51, 52]. In these forums, the discussion points include the lack of evidence and equivocal outcomes of research to support the performance benefits of LCHF but also theoretical constructs around potential benefits to metabolism, muscle, and brain function, inflammatory and oxidative status, and body composition management. Discussion generally targets the potential for “some” [5] athletes to respond to this different dietary approach, with this being promoted to “individuals”, “ultra-endurance athletes”, and “athletes involved in submaximal endurance exercise” [6] while being discouraged for use by athletes involved in “anaerobic performance … or most conditions of competitive athletics” [6]. While there are some suggestions that a larger group of athletes might benefit from an LCHF approach, the general tone is that further investigation of these theories is required [3–6].
2. The apparent caution expressed in peer-reviewed publications is generally not present in other outputs from the same authors. Laybooks [7], web-based information, and social media [8, 9] enthusiastically promote the LCHF dietary approach for a larger group of athletes or athletes in general, with a positive view that this is an evidence-based strategy: “…[in regard to endurance events (60–80 % *V*O_2_max)]: I don’t think there’s much doubt that a low-carb high-fat diet is better. That’s because you have enough fat stores to run for hours and hours and hours. You don’t have many carbohydrate stores to allow you to run for very long. Many of the world’s top endurance athletes have gone low carb, high fat” [8]. The differences between these viewpoints can be confusing, as is the misrepresentation of the physiological requirements of competitive sports (see Sect. 2).
3. The current focus of the LCHF diet movement appears to lie in ketogenic adaptation, or chronic adaptation to a carbohydrate-restricted diet (<50 g/day carbohydrate) with high fat intakes (>80 % of energy). Additionally recommended characteristics include maintenance of moderate protein intake at ~15 % of energy or ~1.5 g/kg/day, with the note that intake should not exceed 25 % of energy intake or ketosis will be suppressed, and the need to ensure adequate intake of sodium and potassium at 3–5 and 2–3 g/day, respectively [6]. Many of the theorized benefits from the LCHF diet are claimed to come from the adaptation to high circulating levels of ketone bodies, which provide an additional fuel source for the brain and muscle as well as achieve other health and functional benefits [5, 6]. The amount of energy that can be provided by ketones as an exercise substrate has been neither calculated nor measured, making it impossible to verify this claim. The time required to achieve optimal adaptation (and, therefore, the period that requires investigation in new studies) is claimed to be at least 2–3 weeks, with at least 1 week required before the feelings of lethargy and reduced exercise capacity abate [5, 6]. With such chronic keto-adaptation, it is considered unnecessary to consume carbohydrate during exercise, or perhaps to consume it in small amounts [5, 6]. As has been discussed in this review, the current evidence for these claims is equivocal and mostly anecdotal. Until or unless further research is undertaken, we are unlikely to resolve any of the current questions and claims. The role of non-ketogenic LCHF diets is not clear.
4. The current literature on LCHF diets is relentless in promoting misunderstanding or misinformation on the current guidelines for athletes in relation to carbohydrate intake in the training or competition diet. These guidelines have been provided in Table 1 to frame the current discussions, and contrast strongly with the information presented by LCHF supporters: “In stark contrast to long-standing dogma in sports nutrition emphasizing the essential need for CHO in all forms of exercise regardless of duration or intensity …” [5]. “Exercise scientists teach that since muscle glycogen utilization occurs at high rates (during high-intensity exercise in CHO-adapted athletes), all athletes must be advised to ingest large amounts of CHO before and during exercise” [3]. As a contributor to the evolution of the current sports nutrition guidelines, which have moved away from a universal approach to any aspect of the athlete’s diet, with particular effort to promote an individualized and periodized approach to both carbohydrate intake and carbohydrate availability during the training phase [53], this author finds such misrepresentation to be a disappointing thread.

## Summary and Future Directions

It would benefit sports nutrition for researchers and practitioners to show mutual respect in recognizing the evolution of new ideas and the replacement of old guidelines with new recommendations [53]. Indeed, modern sports nutrition practitioners teach athletes to manipulate their eating practices to avoid unnecessary and excessive intakes of carbohydrates per se, to optimize training outcomes via modification of the timing, amount and type of carbohydrate-rich foods and drinks to balance periods of low- and high-carbohydrate availability and to adopt well-practiced competition strategies that provide appropriate carbohydrate availability according to the needs and opportunities provided by the event and individual experience [14, 54–57]. It is important to consider insights from research and athlete testimonials to identify different scenarios in which one approach might offer advantages over another or to explain divergent outcomes (Table 5), rather than insist on a single ‘truth’ or solution. Indeed, although there is a continual cry to rid sports nutrition of ‘dogma’ [4], it would seem counterproductive if new ideas were as dogmatic as the old beliefs they seek to replace. This author and others continue to undertake research to evolve and refine the understanding of conditions in which low carbohydrate availability can be tolerated or actually beneficial [58, 59]. However, we also recognize that the benefits of carbohydrate as a substrate for exercise across the full range of exercise intensities via separate pathways [16], the better economy of carbohydrate oxidation versus fat oxidation (ATP produced per L of oxygen combusted) [60], and the potential CNS benefits of mouth sensing of carbohydrate [61] can contribute to optimal sporting performance and should not be shunned simply because of the lure of the size of body fat stores. In other words, there should not be a choice of one fuel source or the other, or ‘black versus white’, but rather a desire to integrate and individualize the various dietary factors that can contribute to optimal sports performance.

**Table 5:** Scenarios or explanations for testimonials/observations of enhanced performance following change to a low-carbohydrate high-fat diet

Scenarios favoring adaptation to LCHF diet	Other explanations for anecdotal reports of performance benefits from switching to LCHF diet
Individuals or events involving prolonged sub-maximal effort where there is no benefit or requirement for higher-intensity pieces Individuals or events in which it is difficult to consume adequate CHO to meet goals for optimal CHO availability (e.g., gastrointestinal upsets, logistical difficulties with accessing supplies during the event) Individuals who are carbohydrate sensitive and likely to be exposed to low CHO availability	Switch to LCHF has been associated with loss of body fat and increase in power-to-mass ratio Previous diet and training were sub-optimal, and switch has been associated with greater training and diet discipline Order effect: natural progress in training and maturation in age and sporting experience Previous program did not include accurate measurement of performance: awareness of performance metrics just commenced Placebo effect/excitement about being part of new idea/culture Athlete is not actually adhering to LCHF diet, due to misunderstanding of its true composition or own ‘tweaking’ activities, such that eating patterns include sufficient CHO around key training sessions and competition to promote high CHO availability

*CHO* carbohydrate, *LCHF* low-carbohydrate high-fat diet

The science and practice of these strategies is still evolving, and indeed, a final comment by this author on the current literature on LCHF diets for sports performance is that another reason for considering it incomplete is that the optimal ‘control’ (or additional intervention) diet has not yet been included in comparisons with fat-adaptation techniques. Future studies should investigate various LCHF strategies in comparison with the evolving model of the ‘carbohydrate-periodized’ training diet, rather than (or as well as) a diet chronically high in carbohydrate availability, to determine the best approaches for different individuals, different goals, and preparation for different sporting events. Considering that athletes might best benefit from a range of options in the dietary tool box is likely to be a better model for optimal sports nutrition than insisting on a single, one-size-fits-all solution.
